# Reactive oxygen species from mitochondria impacts trophoblast fusion and the production of endocrine hormones by syncytiotrophoblasts

**DOI:** 10.1371/journal.pone.0229332

**Published:** 2020-02-24

**Authors:** O’Llenecia S. Walker, Rehginald Ragos, Michael K. Wong, Mohamed Adam, Anson Cheung, Sandeep Raha

**Affiliations:** Department of Pediatrics and the Graduate Program in Medical Sciences, McMaster University, Hamilton, Canada; Shanghai Jiao Tong University, CHINA

## Abstract

The placenta, a tissue that is metabolically active and rich in mitochondria, forms a critical interface between the mother and developing fetus. Oxidative stress within this tissue, derived from the dysregulation of reactive oxygen species (ROS), has been linked to a number of adverse fetal outcomes. While such outcomes have been associated with mitochondrial dysfunction, the causal role of mitochondrial dysfunction and mitochondrially generated ROS in altering the process of placentation remains unclear. In this study, mitochondrial complex I activity was attenuated using 10 nM rotenone to induce cellular oxidative stress by increasing mitochondrial ROS production in the BeWo choriocarcinoma cell line. Increased mitochondrial ROS resulted in a significant decrease in the transcripts which encode for proteins associated with fusion (*GCM1*, *ERVW-1*, and *ERVFRD-1*) resulting in a 5-fold decrease in the percentage of BeWo fusion. This outcome was associated with increased indicators of mitochondrial fragmentation, as determined by decreased expression of MFN2 and OPA1 along with an increase in a marker of mitochondrial fission (DRP1). Importantly, increased mitochondrial ROS also resulted in a 5.0-fold reduction of human placental lactogen (*PL*) and a 4.4-fold reduction of insulin like growth factor 2 (*IGF2*) transcripts; hormones which play an important role in regulating fetal growth. The pre-treatment of rotenone-exposed cells with 5 mM N-acetyl cysteine (NAC) resulted in the prevention of these ROS mediated changes in BeWo function and supports a central role for mitochondrial ROS signaling in the maintenance and function of the materno-fetal interface.

## Introduction

Throughout gestation, the placenta supports the nourishment, growth, and development of the fetus by providing immunological protection and the secretion of various hormones and growth factors necessary for embryonic and fetal survival [1]. The establishment of this critical materno-fetal exchange interface results from the terminal differentiation and fusion of mononucleated villous cytotrophoblasts (CTs) into the multinucleated syncytiotrophoblasts (ST) [1, 2]. Essential to this process is the mitochondrion, an organelle that regulates energy production and serves as the primary source of reactive oxygen species (ROS) [3, 4]. While ROS is an important cellular signal, increased exposure can result in significant damage to mitochondrial function and contribute to overall placental dysfunction [2]. Altered mitochondrial function in the placenta has been associated with various pathological conditions [5] such as intrauterine growth restriction (IUGR). This condition is commonly caused by uteroplacental insufficiency, which in part, may stem from deficiencies in the CT/ST differentiation pathways [6]. Other pregnancy complications, such as preeclampsia (PE) or even pregnancy failure, can stem from perturbations to this differentiation pathway [7]. Perturbed syncytialization will limit the transplacental delivery of critical substrates such as oxygen, glucose and amino acids to the fetus, as well as the removal of fetal waste products. Thus, minor defects in placentation can have profound effects on pregnancy outcome [8]. Importantly, these conditions are often associated with increased oxidative stress [9, 10] in the placenta and result in altered fetal growth. Additional support for the integral role of mitochondrial function in placentation is provided by the observations of Chen and Chan (2010) which demonstrate that mutations in placental mitochondrial fusion genes lead to embryonic lethality [11]. Taken together, the rapid growth and development of the placenta during the early stages of pregnancy makes it susceptible to oxidative stress/damage; a period of vulnerability which is thought to last through to the end of the second trimester [12]. During this period, insults which alter mitochondrial function can potentiate the level of placental oxidative stress and increase the risk of adverse pregnancy outcomes. Mechanistically, the altered intrauterine environment that ensues may affect fetal development by modifying gene expression and the functioning of trophoblasts. Alterations to mitochondrial function can have deleterious effects, particularly in cells that have a high metabolic demand, such as placental trophoblast cells [12]. Trophoblasts have a high demand for ATP [2] which allows for active transport of substrates across the materno-fetal interface and for continued proliferation of the cytotrophoblasts throughout gestation [1].

Mitochondria are dynamic organelles which are involved in a variety of cellular processes including OXPHOS, apoptosis, and cellular stress responses [3]. They participate in these processes and regulate cellular function through calcium signaling, ROS generation and altered mitochondrial morphology resulting from changes to the fission and fusion dynamics. While complex I and III are generally thought of as the primary sources for free radical generation [3], recent evidence suggests that complex II may also contribute to the pool of mitochondrially generated ROS [13]. Rotenone, the most potent member of the rotenoids, enhances ROS formation during forward electron transfer from FeS centres to ubiquinone leading to superoxide production from mitochondrial respiratory chain complex I and can result in increased oxidative stress to cellular components [3]. Therefore, rotenone [3] is a valuable tool to mimic complex 1-associated disorders and initiate mitochondrial production of ROS in a targeted fashion.

Using an *in vitro* model of syncytialization, we hypothesize that mitochondrially-generated ROS, induced by pharmacological inhibition of complex I in placental BeWo cells, leads to reduced BeWo syncytialization, and altered secretory profile of the trophoblast cells. We employed an antioxidant precursor, N-acetyl cysteine (NAC), to assess the role of ROS in mediating many of the adverse effects of excess ROS production in BeWo cells.

## Materials and methods

### Cell culture

The work in this manuscript was conducted in accordance with McMaster University Biosafety Utilization Protocols (BUP-023) and with the approval of the McMaster University Biosafety committee. BeWo cells (ATCC^®^ CCL-98) were grown and maintained in Ham’s F-12K medium supplemented with 10% FBS, 1% penicillin/streptomycin, and 1% L-glutamine, maintained in a humidified atmosphere of 5% CO_2_ at 37°C. For experimental analysis, cells were seeded at 10,000 cells/cm^2^ (approximately 70% confluence). 24 hrs later, cells were treated with epidermal growth factor (EGF; 50ng/mL) to facilitate monolayer formation via its ability to induce proliferation [14, 15]. Following 48 hrs of EGF treatment, the media was supplemented with forskolin (FSK; 50μM) to promote fusion [16, 17], and EGF. The BeWo cells were permitted to differentiate for 48 hrs and then harvested for the analysis outlined below. Rotenone treatment was carried out by exposing BeWo cells to 10 nM rotenone at the same time at FSK/EGF supplementation; in total rotenone treatment lasted for 48 hrs in all cases. The effects of NAC pretreatment on rotenone-mediated ROS generation were investigated by supplementing the media with 5 mM NAC at the same time as EGF addition, 24 hrs following seeding of the cells. Following 48hrs, the media was then supplemented with rotenone, EGF and FSK at the concentrations outlined above. Following 48 hrs of treatment, cells were harvested for immunofluorescence, RNA analysis and Western blot analysis.

### MTS assay (3-(4,5-dimethylthiazol-2-yl)-5-(3-carboxymethoxyphenyl)-2-(4-sulfophenyl)-2H-tetrazolium)

In order to determine concentrations of rotenone that would inhibit complex 1 function without causing overwhelming toxicity, BeWo cells were subcultured into a 96-well plate at a density of 1 x 10^5^ cells/cm^2^ in 100 μL of media. Once a confluency of 70–80% was reached, cells were treated with various concentrations of rotenone. Control wells containing media without cells were allocated to determine background absorbance. Following 48 hours of rotenone exposure, the cells were treated with 20 μL of CellTiter 96^®^ AQ_ueous_ Non-Radioactive Cell Proliferation Assay (Promega, G5421) for 2 hours at 37°C in a humidified, 5% CO_2_ atmosphere. The absorbance was immediately recorded at 490nm using a 96-well plate reader (Miltiskan^®^ Spectrum spectrophotometer; Thermo Scientific, Canada) as per the manufacturer’s instructions. The average 490nm absorbance value from the “no cell” control wells were subtracted from all other absorbance values to yield the corrected absorbance. Further experiments with rotenone were carried out by using the concentration that gave half of the maximal response.

### Lactate dehydrogenase (LDH) assay

As a measure of cell death, plasma membrane integrity was quantified using a LDH assay. Lactate dehydrogenase (LDH) release into culture supernatants was detected spectrophotometrically at 490nm and 680nm, using the Pierce LDH Cytotoxicity Assay Kit (Cat. No. #88953), according to the manufacturer’s recommended protocol. The results are presented as fold increase in the absorbance measured (normalized to untreated cells).

### DCFDA assay (2’,7’-dichlorofluorescin diacetate)

BeWo cells were seeded in a black, clear bottom 96-well microplate at a cell density of approximately 3000 cells per well and supplemented with EGF (50 ng/mL), FSK (50 μM), rotenone (10 nM), and/or NAC (5 mM). The protocol was carried out as per the manufacturer’s instructions (Abcam DCFDA Cellular ROS Detection Assay Kit, ab113851). Using 1X supplemented buffer as the diluent, cells were treated with initial drugs as listed above in a total of 100 μL total volume per well. Tert-Butyl Hydrogen Peroxide (TBHP) solution (100 μM) in 1X supplemented buffer was used as the positive control. After an incubation time of 4 hours, cells were analyzed on a fluorescent plate reader (BioTek Synergy 4) at excitation and emission wavelengths of 485 and 535, respectively. Data were standardized as a percent of control after background (blank wells with media only) subtraction, followed by normalization to total protein content (BCA).

### Total protein extraction

Total protein was isolated following cell lysis using ice-cold radioimmunoprecipitation assay (RIPA) buffer supplemented with protease and phosphatase inhibitor tablets (1 tablet each per 10 mL of RIPA). Cells were further homogenized by sonication for 7–10 pulses at 7 Hz. Samples were centrifuged at 10 000 xg for 5 minutes at 4°C to pellet the cell debris. The supernatant containing total protein was transferred to a fresh tube and placed on ice for immediate BCA analysis or stored at -80°C for later use.

### BCA assay

Protein concentration was determined by using the bicinchoninic assay (BCA; ThermoFisher, Canada) with bovine serum albumin (BSA; 0–2000 μg/mL) as a concentration standard according to manufacturers instructions. Total protein concentration was measured with a 96-well plate reader (Miltiskan^®^ Spectrum spectrophotometer; Thermo Scientific, Canada) at 562nm.

### SDS-PAGE and western blotting

20 μg of total protein was separated on a 10% polyacrylamide gel (unless otherwise stated) and transferred to PVDF membranes. Following the transfer, the membranes were rinsed with tris-buffered saline with tween (TBS-T; TBS with 0.1% Tween) and stained with 1X AMIDO to confirm the efficiency of the transfer. The membranes were then rinsed with water and immersed in an aqueous solution of 0.1 M NaOH for 10–30 seconds, followed by a rinse with water.

PVDF membranes were blocked in 5% skim milk in TBS-T at room temperature for 2 hours. Primary antibodies of interest were then diluted in 5% milk in TBS-T and the membranes were incubated overnight at 4°C. The membranes were washed three times, five minutes each in TBS-T before incubation in secondary horseradish peroxidase-linked donkey anti-rabbit or sheep anti-mouse antibody (1:5000 dilution, GE Healthcare UK) for 1 hour at room temperature. After washing in TBS-T, immunoreactive bands were visualized using an enzyme-linked chemiluminescence detection reagent (Pierce) and visualized using Image Reader LAS-3000IR (Fujifilm). The intensity of the bands were quantified using ImageJ. The ImageJ values generated for the proteins of interest were normalized to GAPDH and/or beta actin on the same membrane.

### Immunofluorescence

BeWo cells were seeded on coverslips and incubated with the corresponding treatments. Cells were washed with DPBS and fixed with 2% paraformaldehyde (PFA) for 10 minutes at room temperature. After fixation, they were washed twice with PBS-T (PBS and 0.01% Tween 20) at room temperature. 10% goat serum with 1% BSA, diluted in PBS-T, was used to block the samples for 2 hours at room temperature. Cells were washed twice with PBS-T. The primary antibody against E-cadherin EP700Y (Abcam) was diluted (1:500) in PBS-T with 0.1% BSA. The primary antibody solution was incubated overnight at 4 °C. Cells were washed twice with PBS-T. A goat anti-rabbit antibody conjugated to AlexaFluor 488 (Abcam) was diluted in PBS-T with 0.1% BSA to a 1:100 dilution. Cells were incubated with the secondary antibody solution for 2 hours at room temperature in the dark. After washing twice with PBS-T, cells were incubated with a DAPI solution (1.5 ug/mL, diluted in PBS-T and 0.1% BSA, Santa Cruz) for 5 minutes in the dark. Cells were washed twice with PBS-T and mounted onto microscope slides with Fluoromount^™^ (Diagnostic Biosystems Inc., USA). Coverslips were imaged at 200X magnification with a Nikon Eclipse Ti-E (Nikon Instruments Inc., USA). Five non-overlapping fields of view were captured per sample. Total fusion percentage was calculated as follows: (total number of nuclei in fused cells/total number of nuclei) *100%. Fused cells were counted as cells that had more than one nucleus per continuous membrane as visualized by E-cadherin staining.

### RNA extraction and RT-PCR

Cells grown/treated on 12-well plates were lysed with 500 μL of ice-cold TRIzol^™^ reagent (Thermo Fisher Scientific, Canada) and homogenized by trituration and left to incubate at room temperature for 5 minutes. After 5 minutes, RNA was either immediately isolated or stored at -80°C. RNA was isolated using the Direct-zol^™^ RNA kit (Zymo Research, USA) as per the manufacturer’s instructions. 500 ng of the resulting RNA was converted to cDNA with the High-Capacity cDNA Reverse Transcription Kit (Applied Biosystems^™^, Canada) as per the manufacturer’s instructions. RT-PCR was performed (CFX384 Touch^™^ Real-Time PCR Detection System, Bio-Rad, Canada) by adding 7 μL of primer mix (5 μL SYBR Green, 1.5 μL primer (2.5 μM of forward and reverse primers), 0.5 μL ddH2O) and 3 μL of 1:10 diluted cDNA. All genes and their respective primer sequences are listed in Table 1. Fold change mRNA expression was quantified using ΔΔCt analysis, normalized to housekeeping gene (18S) then expressed as the relative fold change to the vehicle control sample expression.

**Table 1:** Primer sequences of human genes analyzed via RT-PCR.

Gene	Forward (5’→3’)	Reverse (5’ → 3’)
*18S*	CACGCCACAAGATCCCA	AAGTGACGCAGCCCTCTATG
*CGα*	GCAGGATTGCCCAGAATGC	TCTTGGACCTTAGTGGAGTGG
*CGβ*	ACCCCTTGACCTGTGAT	CTTTATTGTGGGAGGATCGG
*CuZnSOD*	AAAGATGGTGTGGCCGATGT	CAAGCCAAACGACTTCCAGC
*DRP1*	AAACTTCGGAGCTATGCGGT	AGGTTCGCCCAAAAGTCTCA
*GCM1*	CCTCTGAAGCTCATCCCTTGC	ATCATGCTCTCCCTTTGACTGG
*ERVW-1*	GTTAATGACATCAAAGGCACCC	CCCCATCTCAACAGGAAAACC
*ERVFRD-1*	GCCTACCGCCATCCTGATTT	GCTGTCCCTGGTGTTTCAGT
*HSP60*	GAAGGCATGAAGTTTGATCG	TTCAAGAGCAGGTACAATGG
*HSP70*	GGAGTTCAAGAGAAAACACAAG	AAGTCGATGCCCTCAAAC
*IGF2*	GCCAATGGGGAAGTCGATGCTGG	GAGGCTGCAGGATGGTGGCG
*MFN1*	TTGGAGCGGAGACTTAGCAT	GCCTTCTTAGCCAGCACAAAG
*MFN2*	CACAAGGTGAGTGAGCGTCT	ACCAGGAAGCTGGTACAACG
*MnSOD*	GCTCCGGTTTTGGGGTATCT	GATCTGCGCGTTGATGTGAG
*OPA1*	GCTCTGCATACATCTGAAGAACA	AGAGGCTGGACAAAAGACGTT
*PL*	GCCATTGACACCTACCAG	GATTTCTGTTGCGTTTCCTC

### Statistical analyses

Data are presented as the mean ± S.E.M. of three independent experiments. For comparisons between treatment groups, one-way ANOVA or two-way ANOVA followed by Bonferroni’s *post hoc* test was performed using the GraphPad Prism software version 6.0. Differences were considered significant at P < 0.05.

## Results

### Inhibition of complex I results in increased ROS formation, concomitant with the induction of cellular stress responses and upregulation of antioxidant defences

**Fig 1:**
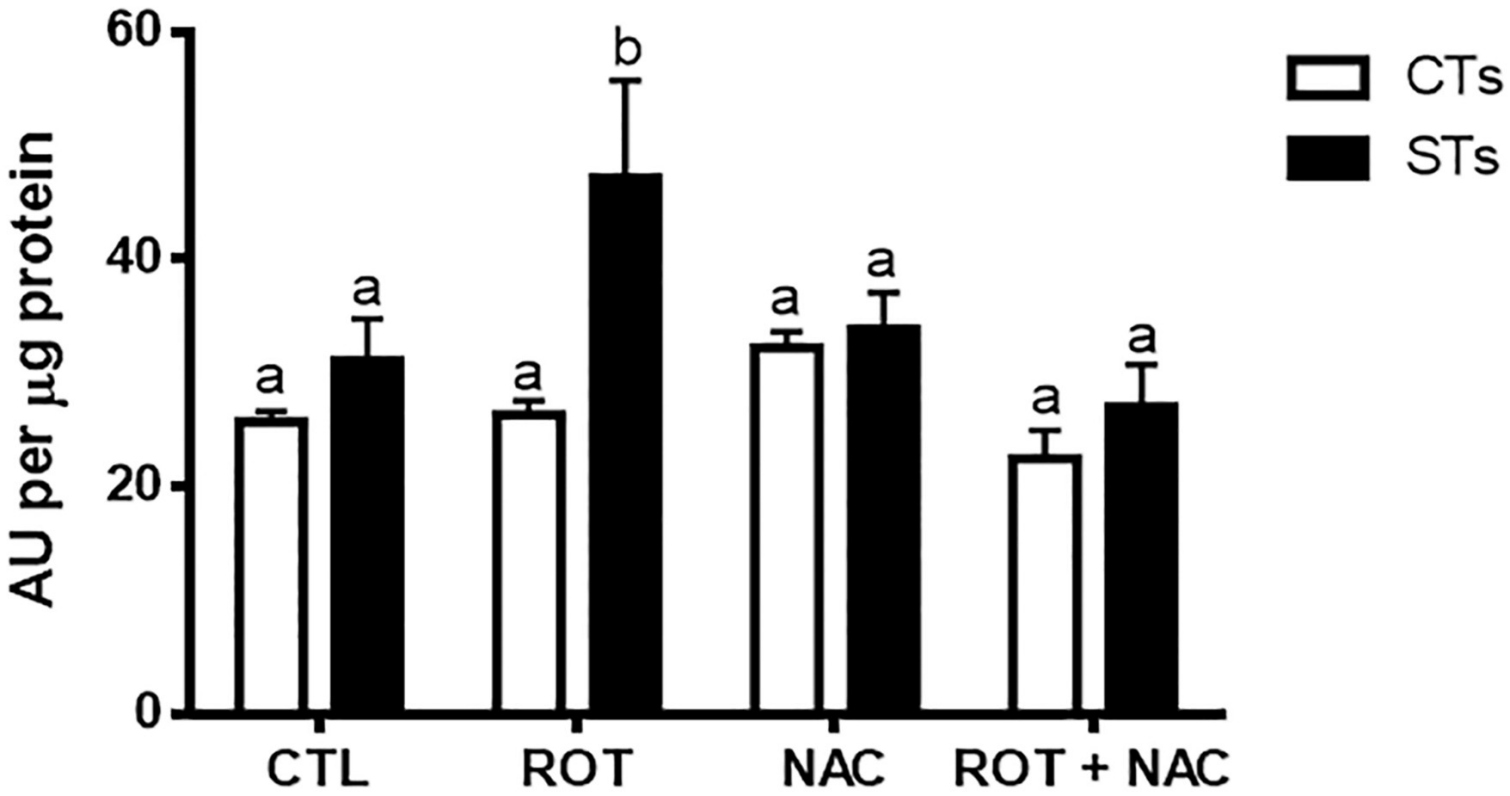
Rotenone induces intracellular ROS production in syncytiotrophoblasts. A DCFDA assay was performed to determine intracellular ROS levels following rotenone and/or NAC exposure. Significance was determined by a two-way ANOVA, followed by a Bonferroni post hoc test. Results were normalized to the protein content of cell lysates via the BCA assay, as described by Masaki et al (2009), which enables the standardization of ROS production as a function of cell number (protein level). The data represents the mean ± SEM (n = 3). Bars with different letters differ significantly at P < 0.05. CTL = control, ROT = rotenone, NAC = N-acetyl cysteine, CT = cytotrophoblasts, ST—syncytiotrophoblasts. AU = arbitrary fluorescence units.

**Fig 2:**
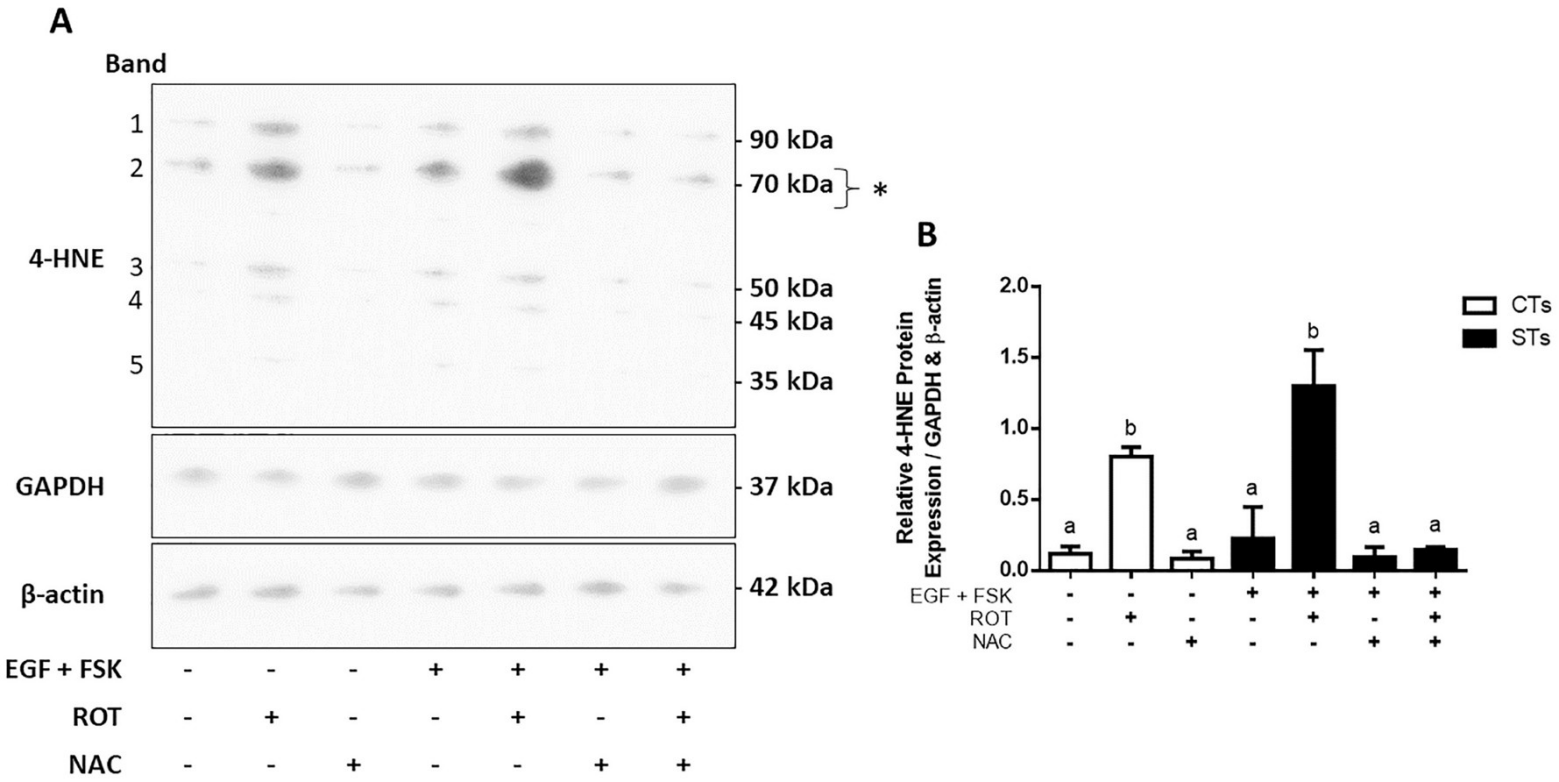
Rotenone-induced mitochondrial complex I inhibition promotes lipid peroxidation in BeWo trophoblast cells. Panel A: Five major protein bands with molecular weights of approximately 90, 70, 50, 45, and 35 kDa showed immunoreactivity for 4-HNE modifications in control and treated trophoblast cells from whole cell lysates. Panel B: Quantification of the density of the five major protein bands for each treatment group are shown, normalized to GAPDH and β-actin. The asterisk (*) indicates a ~70 kDa band of proteins which show greater sensitivity to oxidative damage. Significant differences were determined by a one-way ANOVA followed by a Bonferroni post hoc test. Results are mean ± SEM (n = 3). Bars with different letters differ significantly at P < 0.01 (B). EGF = epidermal growth factor, FSK = forskolin, ROT = rotenone, NAC = N-acetyl cysteine.

We first sought to determine the concentration of rotenone which would allow for complex I inhibition without severely compromising cell viability using an MTS assay (S1 Fig, panels A and B). An IC_50_ value of 10 nM was determined, in the MTS assay, for undifferentiated and differentiated BeWo cells. We also carried out an LDH assay (S1 Fig, panels C and D) at these same concentrations. Cell death at 10 nM was not significantly different from untreated cells, but did increase significantly following treatment with 100 nM rotenone. Since inhibition of complex I is known to increase oxidative stress [3], we performed a ROS assay to determine intracellular ROS levels. ROS levels were increased by 1.5-fold in the ST (differentiated BeWo cells) population (P < 0.05), while the levels remained relatively unchanged across the CT (undifferentiated BeWo) cells (Fig 1). We next assessed whether there was a change in oxidative damage by quantifying levels of 4-hydroxynonenal (4-HNE); a well-established marker of oxidative lipid damage [18]. In response to increased levels of ROS demonstrated in Fig 1, rotenone treatment of BeWo cells resulted in increased levels of 4-HNE in both the CT (10.1-fold) and ST cells (4.8-fold) (Fig 2; P < 0.01). Importantly, pre-treatment with NAC conferred protection against increased intracellular ROS levels (Fig 1) and prevented an increase in 4-HNE production in the ST population (Fig 2).

**Fig 3:**
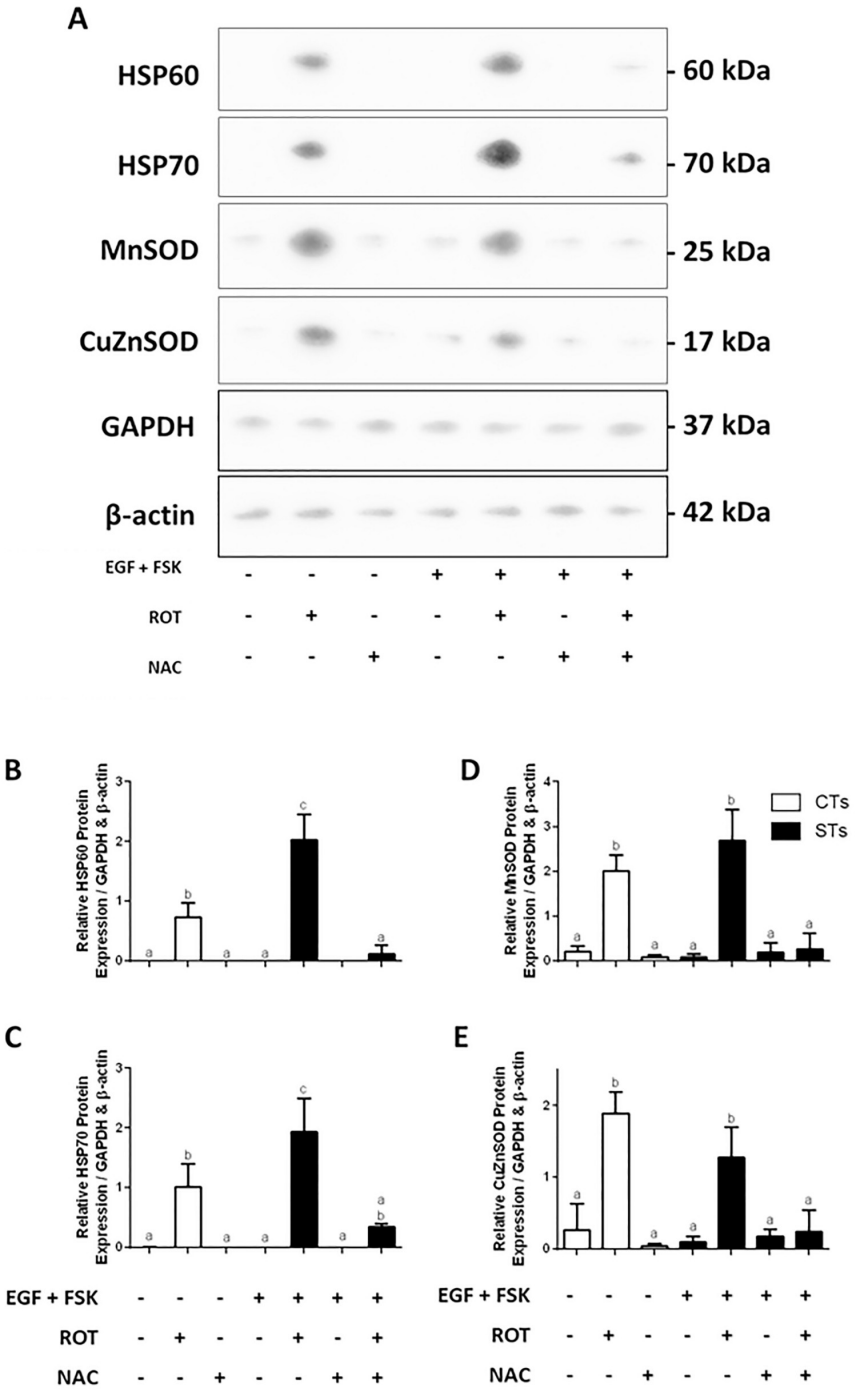
Rotenone treatment of BeWo cells results in the induction of cellular stress response proteins and antioxidant defense enzymes. Panel A: Western blot analyses of HSP60, HSP70, MnSOD, and CuZnSOD from whole cell lysates from BeWo trophoblast cells. Panels B-E: Summary histograms representing the densitometric measurements of Western blots of HSP60, HSP70, MnSOD, and CuZnSOD protein expressions. Data are presented as mean ± SEM, n = 3. Bars with different letters differ significantly at P < 0.05 (B, C), P < 0.001 (D), P < 0.01 (E).

Oxidative damage to cells can result in one of two opposing responses: apoptosis to remove damaged cells, or a stress response as an attempt to prevent damage and facilitate recovery [19]. Since complex I inhibition did not result in significant cell death (MTS and LDH assay in S1 Fig), we decided to examine the induction of stress responses in the BeWo cells. We examined protein biomarkers associated with management of cellular stress such as heat shock proteins 60 and 70 (HSP60, HSP70) and antioxidant enzymes manganese superoxide dismutase (MnSOD) and copper zinc superoxide dismutase (CuZnSOD) (Fig 3). In all cases, exposure to 10 nM rotenone for 48 hrs increased the expression of HSP60, HSP70, MnSOD or CuZnSOD (Fig 3. Panels B-E, respectively). In both the CT and ST trophoblasts, rotenone exposure resulted in induced stress responses and increased the relative levels of MnSOD and CuZnSOD (Fig 3) protein expression. The CT and ST cells showed a significant increase in the expression of HSP60 (Fig 3A and 3B) and HSP70 (Fig 3A and 3C) protein (P < 0.01 for CT, P < 0.0001 for ST). Furthermore, there was a significant upregulation of MnSOD (P < 0.001 for CT; P < 0.0001 for ST) and CuZnSOD (P < 0.01 for CT; P < 0.0001 for ST) in response to the rotenone-induced ROS production in both the CT and ST populations (Fig 3A, 3D and 3E). These rotenone-mediated changes were all normalized in the presence of 5mM NAC. Furthermore, the changes in the expression of the mRNA’s encoding these proteins (S2 Fig) also paralleled what is observed in the Western blots shown in Fig 3.

### Mitochondrial function is important for trophoblast differentiation/syncytialization

**Fig 4:**
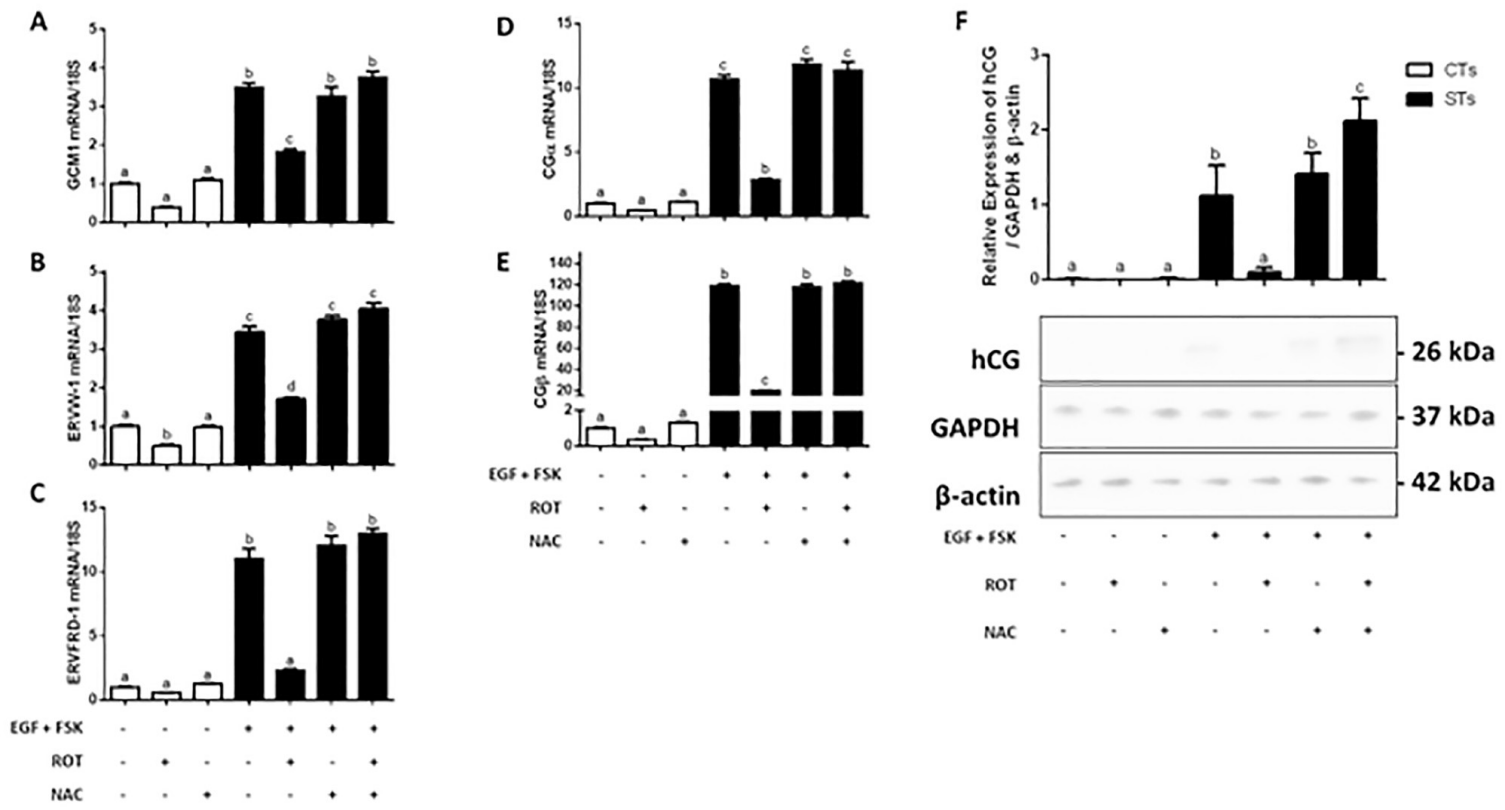
Transcriptional and translational markers of syncytialization and biochemical differentiation are significantly decreased by rotenone. Differentiated BeWo (+FSK, +EGF) or undifferentiated BeWo cells were treated with rotenone, NAC or in combination. The following concentrations were used and administered to cells as described in Methods: DMSO (0.01%) vehicle control, FSK (50 μM), EGF (50 ng/mL), NAC (5 mM) and rotenone (10 nM). Total RNA was isolated from the cells and analyzed by RT-PCR (500 ng) with 18S used as the housekeeping gene. Summary histograms of relative *GCM1* (A), *ERVW-1* (B), *ERVFRD-1* (C), *CG*_*α*_ (D), and *CG*_*β*_ (E) mRNA expression in each treatment group normalized to 18S, then compared to the gene in the vehicle control. Gene fold changes are indicated in the histograms. (F) Quantification of total hCG protein expression from whole cell lysates and a representative Western blot are shown, with GAPDH and β-actin used as the loading controls. Significant differences were determined by a one-way ANOVA, followed by a Bonferroni post hoc test. Data are presented as means ± SEM (n = 3). Bars with different letters differ significantly at P < 0.01 (A), P < 0.05 (B, D), P < 0.0001 (C, E), P < 0.001 (F).

In response to rotenone exposure, CT and ST cells showed a 2.1- and 2.0-fold reduction, respectively, in transcript levels of *ERVW-1* (Fig 4B; P < 0.05). However, only ST cells demonstrated a 1.9-, 4.8-, 3.8-, and 5.9-fold reduction in *GCM1* (Fig 4A; P < 0.01), *ERVFRD-1*, *CGα* and *CGβ* transcript levels (Fig 4C–4E; P < 0.0001, P < 0.05, P < 0.0001) respectively. Upon examination of total hCG protein expression (Fig 4F), similar trends followed, with rotenone treatment reducing the expression of hCG in the ST cells by 12.3-fold (P < 0.001). While NAC pretreatment mitigated the rotenone-mediated decreases in *GCM1*, *ERW-1*, *ERVFRD-1*, CG_*α*_ and CG_*β*_ transcripts (Fig 4, panels A-E), it potentiated the level of hCG protein when compared to ST cells not exposed to rotenone (Fig 4F; P < 0.05).

### Mitochondrial ROS production disrupts basal mitochondrial fission/fusion and reduces trophoblast fusion

**Fig 5:**
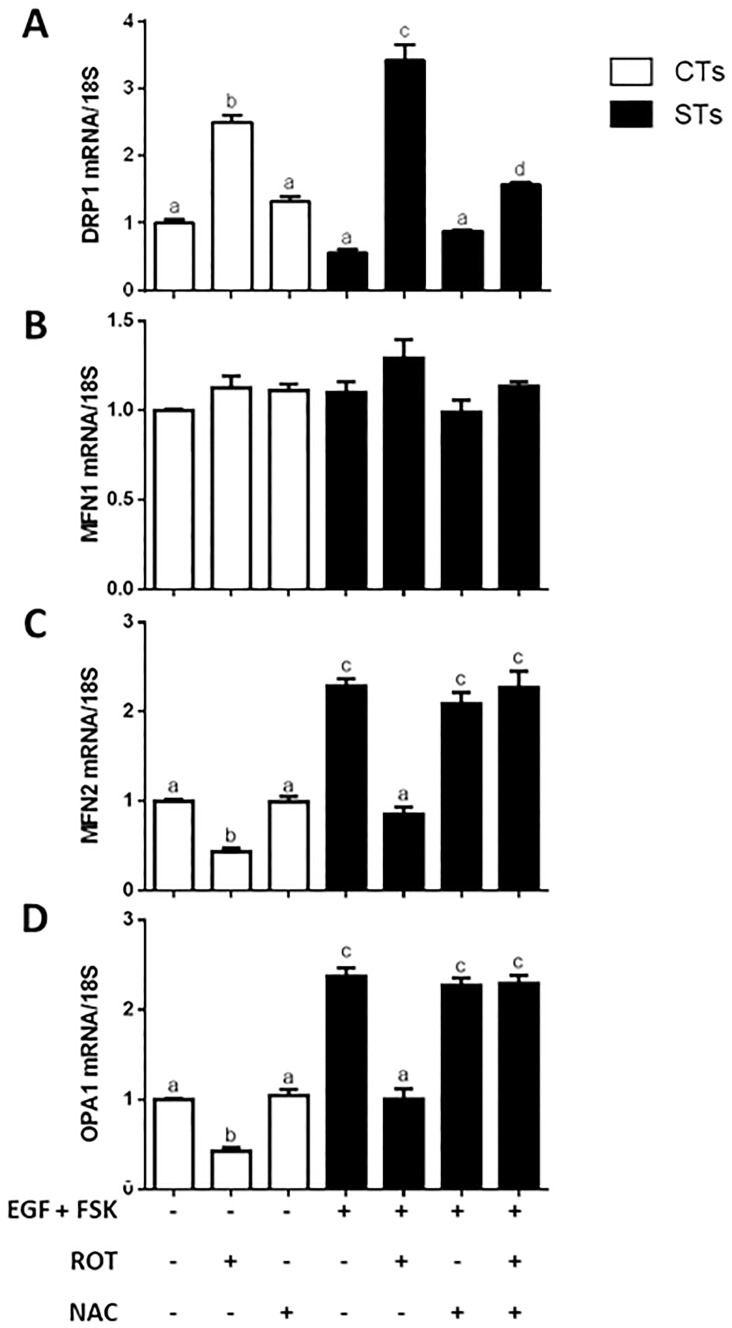
Rotenone treatment of BeWo cells results in altered mRNA expression of the indicators mitochondrial fission and fusion. Total RNA was isolated from the cells and analyzed by RT-PCR (500 ng) with 18S used as the housekeeping gene. Summary histograms of relative DRP1 (A), MFN1 (B), MFN2 (C), and OPA1 (D) mRNA expression in each treatment group normalized to 18S, then compared to the gene in the vehicle control group. Significant differences were determined by a one-way ANOVA, followed by a Bonferroni post hoc test. Data are presented as means ± SEM (n = 3). Bars with different letters differ significantly at P < 0.05 (A, C), P = 0.0515 (ns) (B), P < 0.01 (D).

**Fig 6:**
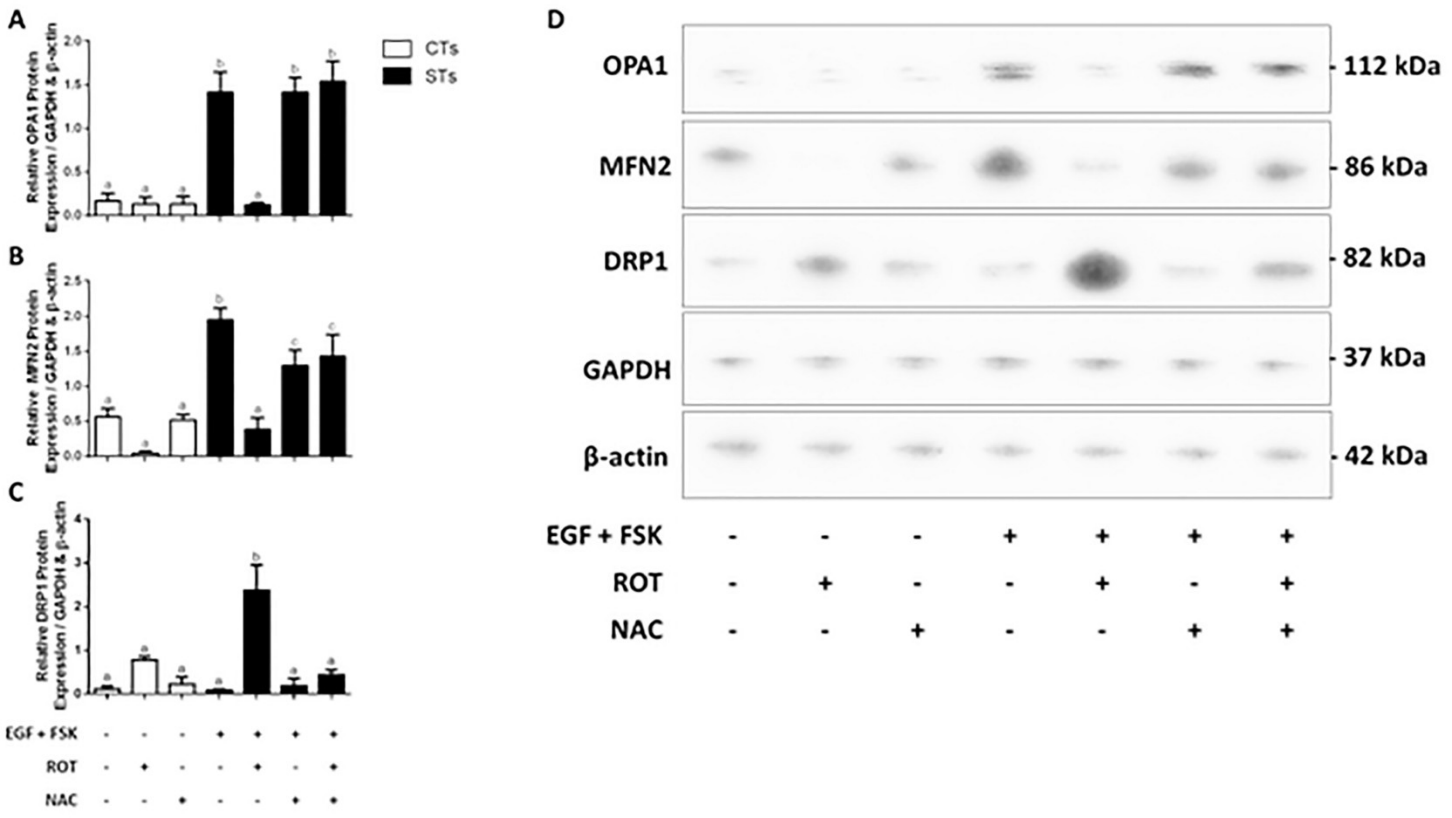
Rotenone treatment alters the expression of proteins responsible for mitochondrial morphology. Total protein was isolated from BeWo cells and analyzed by Western blot (20μg) with GAPDH and β-actin used as loading controls. Summary histograms of relative OPA1 (A), MFN2 (B), and DRP1 (C) of relative band density in each treatment group normalized to total GAPDH and β-actin. Significant differences were determined by a one-way ANOVA, followed by a Bonferroni post hoc test. Data are presented as means ± SEM (n = 3). Bars with different letters differ significantly at P < 0.0001 (A, C), P < 0.05 (B).

Both CT and ST cells exhibited increased levels of *DRP1* transcript levels (Fig 5A; P < 0.05), by 2.5- and 6.1-fold, respectively in response to rotenone treatment. Concomitantly, there was a 2-fold decrease in *MFN2* and *OPA1* transcript levels (Fig 5C and 5D; P < 0.05, P < 0.01) respectively. No change was observed in *MFN1* transcript level (Fig 5B). There were no statistically significant changes in the mitochondrial morphology proteins OPA1, MFN2, nor DRP1 (Fig 6A–6C, respectively) in the CT population of BeWo cells. However, upon rotenone treatment, the levels of OPA1 and MFN2 protein expressions were markedly reduced (P < 0.0001, P < 0.05, respectively) along with a 26.5-fold increase in DRP1 expression (Fig 6) in the ST cells. Importantly, pretreatment of rotenone-exposed cells with NAC resulted in transcript (Fig 5C and 5D) and protein (Fig 6A, 6B and 6D) levels of OPA1 and MFN2 that were similar to that observed in the vehicle treated cells. Increases in *DRP1* transcript were partially protected against in response to NAC pre-treatment (Fig 5A; P < 0.05), however, this effect was not evident at the level of protein expression (Fig 6D).

**Fig 7:**
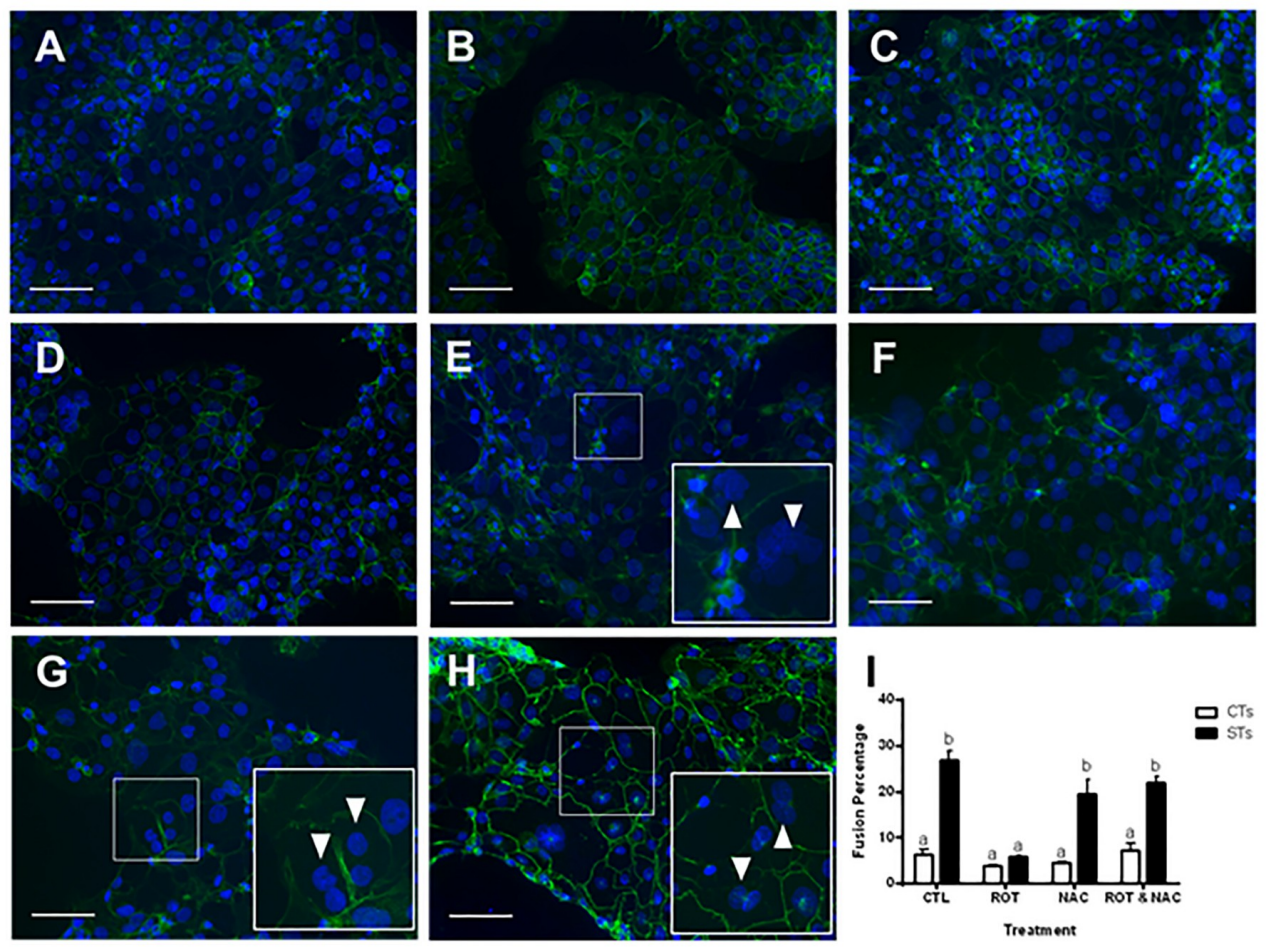
Rotenone-treatment of BeWo prevents reduces cellular fusion. BeWo cells showing immunofluorescent staining (A-H) for E-cadherin distribution at the cell membrane using FITC-conjugated secondary antibody (green) and counterstained with 4’, 6-diamidino-2-phenylindole (DAPI) identifying the nuclei (blue). Representative fluorescent microscopy images (20X) are shown (A-H), selected from five random, non-overlapping regions per treatment group (n = 3); scale bars indicate 100 μm. A = CT CTL, B = CT ROT, C = CT NAC, D = CT ROT + NAC, E = ST CTL, F = ST ROT, G = ST NAC, H = ST ROT + NAC. Insets represent close up magnifications of E-cadherin localization with depiction of multinucleated syncytiotrophoblasts (arrowheads). Cell counts were performed by two blinded researchers, the average of which were calculated to determine the fusion percentage across all groups (I). Significant differences were determined by a two-way ANOVA, followed by a Bonferroni post hoc test. Data are presented as means ± SEM (n = 3). Bars with different letters differ significantly at P < 0.001 (I).

Rotenone treatment of BeWo cells resulted in a reduction in cellular fusion (Fig 7), as evidenced by greater disruption in the continuity of the cell boundaries stained by E-cadherin (green) and the increase in the number of boundaries containing single nuclei, stained by DAPI (blue) (Fig 7B for CTs and Fig 7F for STs). Importantly, pre-treatment with NAC, in the presence of rotenone, allowed the BeWo cells to differentiate and fuse into multinucleated cells (Fig 7H and 7I) to the same extent as the vehicle treated cells.

### Predictors of fetal growth are altered in response to mitochondrial dysfunction in placental cells

**Fig 8:**
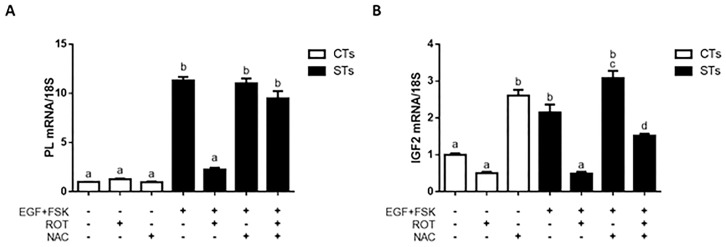
Predictors of fetal growth are impacted by rotenone-treatment of BeWo cells. BeWo cells were treated as labelled. The following concentrations were used and administered to cells as described in Methods: DMSO (0.01%) vehicle control, FSK (50 μM), EGF (50 ng/mL), NAC (5 mM) and rotenone (10 nM). Total RNA was isolated from the cells and analyzed by RT-PCR (500 ng) with 18S used as the housekeeping gene. Summary histograms of relative mRNA expression in each treatment group normalized to 18S, then compared to the gene in the vehicle control. Gene fold changes are indicated in the histograms. Panel A: *hPL* transcript. Panel B: I*GF2* transcript. Data are presented as mean ± SEM, n = 3. Significant differences were determined by a one-way ANOVA, followed by a Bonferroni post hoc test. Bars with different letters differ significantly at P < 0.0001 (A), P < 0.01 (B).

Insulin-like growth factor 2 (IGF-2) [20] and human placental lactogen (hPL) [21] are known to play a role in determining placental nutrient supply and subsequent fetal growth [20]. We quantified the expression of the genes encoding hPL and IGF2 in differentiated and undifferentiated BeWo cells treated with (i) rotenone (ii) NAC (iii) both rotenone and NAC. hPL and IGF-2 expression were not affected in CT cells treated with rotenone (Fig 8A and 8B). Interestingly, NAC treatment produced a 2.5-fold increase in *IGF2* in the CT population (Fig 8B, P < 0.0001). Rotenone treatment of ST cells reduced the level of *PL* by 5.0-fold (Fig 8A) and *IGF2* by 4.4 fold (Fig 8B) (P < 0.0001), an effect which was fully and partially protected against by NAC pre-treatment regarding the expression of *PL* and *IGF2*, respectively.

## Discussion

Fetal growth depends on placental function. Once established, the human placenta mediates all of the physiological exchanges between the mother and her fetus [12]. As an active endocrine organ, it is rich in mitochondria [12, 22], which are required to support the metabolic demands of protein and steroid hormone production. Previous work has demonstrated that mitochondria undergo morphological and functional changes upon fusion of the placental trophoblast cells [23–25]. When compared to mitochondria of the cytotrophoblast population, syncytiotrophoblast mitochondria are smaller, contain vesicular cristae rather than lamellar cristae, and accumulate P450_SCC_, which is a mitochondrial enzyme responsible for catalyzing the first step in progesterone production [23]. These structural differences may result in differential changes to mitochondrial signaling, which may be implicated in altered trophoblast fusion and differentiation programs. Indeed, altered mitochondrial function has been associated with placental pathologies that also demonstrate impaired trophoblast fusion [25–27]. Placentae from PE pregnancies display ultrastructural changes that affect the flow of electrons through the mitochondrial electron transport chain, potentiating excessive ROS production or oxidative stress [26], whereas placentae from IUGR pregnancies demonstrate altered mitochondrial biogenesis [27]. Although the mitochondria are differentially altered in placentae of PE and IUGR pregnancies, both disrupt normal mitochondrial signaling possibly governing placental development. Despite the evidence of mitochondrial dysfunction observed in placental pathologies in the published literature, the exact mechanisms of how it may impair placental development remain elusive. We suggest that alterations to the mitochondria may disrupt the mitochondrial ROS signaling implicated in trophoblast fusion and differentiation processes, ultimately leading to unfavourable clinical manifestations. To demonstrate a link between mitochondrial function and trophoblast fusion, Poidatz *et al*. (2015) administered mitochondrial inhibitors to induce mitochondrial dysfunction in primary cytotrophoblasts *in vitro* [2]. Upon administration, cells demonstrated decreased syncytin mRNA expression and endocrine secretion, which suggest that trophoblast fusion requires fully functional respiratory chain activity [2]. However, the mechanisms by which impaired mitochondrial signaling inhibits trophoblast fusion and differentiation remains to be elucidated. This study investigated how mitochondrially produced ROS, by pharmacologically inhibiting complex I with rotenone, may be implicated in the impaired fusion process. [28].

Oxidative stress is largely implicated in reproductive failures, including but not limited to infertility, miscarriage, endometriosis, pre-term labour, and PE [29]. Furthermore, excess ROS can trigger pathological events in the placenta, embryo, and fetus [30]. Defense mechanisms against free radical production increases as pregnancy progresses [12]. The initial response to oxidative stress is to upregulate defense systems such as HSPs [19] and antioxidant enzymes [12]. HSPs are classically thought of as chaperones that prevent misfolding of proteins in response to various stressors, and to facilitate their refolding and renaturation [19]. In response to harmful stimuli, HSPs are capable of modulating pro-apoptotic signaling pathways to promote survival of the cell [19]. Antioxidant enzymes, in particular the superoxide dismutases, have been shown to increase as gestation progresses, as evidenced from placental preparations at early, midgestation and at term [12], along with free radical scavengers such as glutathione; a response that is likely due to increased O_2_^-^ as pregnancy progresses [12]. Several studies have generated ROS using partial inhibition of complex 1 [2, 31–33]. In our study, though BeWo cells were subjected to mitochondrial dysfunction via rotenone-induced complex I inhibition, the CT cells displayed no significant change in relative ROS levels, while the ST cells exposed to rotenone showed a significant increase in relative ROS levels. This could be explained by our observation that the antioxidant enzymes MnSOD and CuZnSOD were equally upregulated in both the CT and ST cells in response to partial mitochondrial inhibition at complex I by rotenone, while the stress response was significantly greater in the ST population, as evidenced by the greater expression of HSPs in the ST cells. This may carry the implication that the ST cells are more sensitive to changes in their oxidative status, as terminal differentiation into STs involves structural and functional changes to the mitochondria [23, 24]. Indeed, it is reported that placental STs are very sensitive to oxidative stress, in part because they form the lining the placenta and must react to the dynamic changes in maternal blood oxygen concentrations [34]. Contributory to this observation may be the finding that ST cells contain fewer antioxidant enzymes relative to other placental cells, for reasons that have yet to be elucidated [34]. Moreover, research has demonstrated that these two trophoblast subpopulations display different nuclear [35] and mitochondrial [2] morphologies, which may further underscore their differing functions and sensitivities to oxidative perturbations.

Mitochondria are dynamic organelles that continually undergo fusion and fission thus having variable morphological features which are intimately associated with adaptive and functional alterations. Mitochondria participate in key cellular functions and thus affect the processes of most major chronic diseases, aging, and oxidative tissue injury [3]. In this study, the effect of rotenone treatment on differentiating BeWo cells and the associated fusion/fission events was explored in the context of trophoblastic syncytialization. We examined the expression of the mitochondrial pro-fusion markers MFN1, MFN2 and OPA1, along with the pro-fission marker DRP1. Localized to the outer mitochondrial membrane (OMM), MFN1 and MFN2 have been identified as critical for the fusion of the OMM, while OPA1, is localized to and mediates the fusion of the inner mitochondrial membrane (IMM) [36]. Inhibition of fusion in mammalian cells has been associated with loss of mitochondrial respiratory function [37]. Indeed, upon rotenone treatment of BeWo cells, we demonstrate a marked reduction in the expression of MFN2 and OPA1, which is indicative of increased mitochondrial fission. Interestingly, we observed no change in the expression of MFN1 transcript in response to rotenone treatment of BeWo cells. Although both MFN1 and MFN2 are required for the maintenance of normal mitochondrial morphology, it may be that MFN2 plays a more direct role in maintaining the OMM. Indeed, Chen *et al* (2003) have demonstrated that cellular overexpression of one mitofusin while the other is made experimentally deficient is sufficient to restore mitochondrial morphology by promoting fusion [38]. Although the MFN complexes are involved in mitochondrial fusion, they may also serve distinct functions [38]. Largely localized in the cytosol is the opposing dynamin related protein DRP1 which, upon mitochondrial recruitment, promotes OMM and IMM constriction during the process of mitochondrial fission [36, 39]. While some DRP is localized to the mitochondria, likely marking sites of future fission [36], ROS may induce acute recruitment of DRP1 to the mitochondria to orchestrate the process of fragmentation, an effect which was protected against in the ST cells by pre-treatment with NAC.

NAC is known as a ROS scavenger and is used in various clinical applications [40]. In this study, we utilized NAC, an acetylated form of L-cysteine and a substrate for glutathione synthesis, to protect human placental trophoblast cells from excessive ROS-induced damage in an *in vitro* setting. It serves as a powerful antioxidant and has proven useful in diseases which are subject to excessive free radical production (reviewed in Mokhtari *et al*) [40]. Importantly, there are no known detrimental effects of NAC on maternal or fetal metabolism [40]. In this study, we pre-treated BeWo cells with NAC, then selectively reduced electron transport at complex I to determine if we could protect the trophoblast cells from exposure to excessive free radical formation. Our observations demonstrate that perturbing mitochondrial function by interfering with electron trafficking is a detriment to the process of syncytialization. Importantly, the fusogenic potential of the trophoblast cells were preserved upon pretreatment with NAC, protecting the cells from the damaging effects of increased mitochondrial ROS. Interestingly, the CT cells showed a 2.5-fold increase in IGF2 transcript upon NAC treatment. This may be explained, in part, by the insulin sensitizing effect of NAC [40]. Since IGFs promote growth [1], it is logical that they are nutritionally regulated, with their concentrations serving as an indicator of the availability of substrates from the maternal diet, the availability of which will be limited with placental insufficiency. The greatest inference derived from our study is that of the negative effect of mitochondrial dysfunction and increased ROS production on the expression profile of transcripts and proteins which are implicated in the promotion of normal fetal growth. Various hormones are secreted by placental trophoblasts which are crucial for maintaining the viability of the fetus. Some of these hormones have been specifically linked to fetal growth, as they affect aspects of maternal and fetal metabolism [41]. Two key predictors assessed in our study were the transcript levels of IGF2 and hPL.

Insulin-like growth factor (IGF) has a fundamental role in the control of placental and fetal growth. IGF not only stimulates production of glucose and amino acids, but it also facilitates trophoblast proliferation and differentiation [42], thus promoting proper endocrine function. Increasing throughout gestation, progesterone inhibits insulin-like growth factor binding protein-1 (IGFBP-1), thus allowing for an increase in the bioavailability of IGFs to the growing embryo/fetus [43]. Rotenone treatment of BeWo cells resulted in a marked reduction in IGF2 transcript levels. This suggests the increased trophoblastic oxidative stress may, through the dysregulation of IGF2, may contribute to reduced fetal growth. Mechanistically, an increase in IGF is required to stimulate transplacental passage of nutrients, particularly in the third trimester [42]. Furthermore, the action of IGFs are, at least in part, modulated by hPL, which is secreted by the ST cells [16]. Acting with placental growth hormone, hPL is involved in mediating maternal and fetal amino acid, carbohydrate and lipid metabolism [44]. Though able to stimulate the increase in the bioavailability of IGFs, it is not fully known how hPL exerts its somatogenic effects on the fetus [44]. In our hands, increased mitochondrial ROS production, significantly suppressed two key predictors of fetal growth (IGF2 and hPL), effects which were protected against by pretreatment with an antioxidant.

Functionally, rotenone treatment during the BeWo syncytialization process resulted in impaired cell fusion, reduced *GCM1*, *ERVW-1*, and *ERVFRD-1* transcript expression, along with reduced β-hCG secretion. Importantly, our findings reveal the effect of NAC pretreatment protects trophoblasts from the effects of excessive mitochondrial ROS and restored the expression of fusogenic genes and proteins. Other intracellular signaling pathways, such as ER stress and immune signalling, may also contribute to mediating the fusogenic pathways of trophoblast cells as they are known to impact mitochondrial function [45–48].

One important limitation when examining the dynamic trophoblast fusion process is that there is always a mix of differentiating cytotrophoblasts and syncytiotrophoblasts [49]. Therefore, our genomic data reflects changes in both populations and does not detract from our conclusion that mitochondrially produced free radicals significantly attenuates the fusion process.

Taken together, our work demonstrates the importance of mitochondrial health in trophoblast fusion. Thus, pathologies or drugs that involve the mitochondria as a target for their mechanism of action may also put pregnancies at risk for developing placental pathologies. Since ROS signaling is an important part of pregnancy, understanding its regulation and its relationship to fetal growth and development will provide important insights into how placental oxidative stress impacts post-natal health of the fetus.

## Supporting information

S1 Fig 10 nM rotenone negatively impacts proliferation of undifferentiated (CT) and differentiated (ST) BeWo cells without induction of cell membrane damage (A–D). The cells were treated with rotenone as indicated. MTS data in panels A, B. LDH data in panels C, D. Each data point represents the mean ± S.E.M. of 3 replicates. Significant differences were determined by a one-way ANOVA followed by a Bonferroni post hoc test. Points with different letters differ significantly at P < 0.0001 (A-C) P < 0.001 (D) from control. Further experiments with rotenone were carried out by using the concentration that gave half of the maximum response, i.e. the point at which MTS values were reduced by 50%, thus successfully inhibiting RCC1 without causing overwhelming toxicity to the cells (C, D).
(PNG)

S2 Fig Altered transcript expression of HSP60, HSP70, MnSOD and CuZnSOD in rotenone treated BeWo cells. Total RNA was isolated from the cells and analyzed by RT-PCR (500 ng) with 18S used as the housekeeping gene. (A—D) Summary histograms of relative MnSOD (A), CuZnSOD (B), HSP60 (C), and HSP70 (D) mRNA expression in each treatment group normalized to 18S, then compared to the gene in the vehicle control group. Significant differences were determined by a one-way ANOVA, followed by a Bonferroni post hoc test. Data are presented as mean ± SEM, n = 3. Bars with different letters differ significantly at P < 0.0001 (A), P < 0.001 (B), P < 0.01 (C), P <0.05 (D).
(PNG)
