# Supplementary figures and images for: Reactive oxygen species from mitochondria impacts trophoblast fusion and the production of endocrine hormones by syncytiotrophoblasts
Source: PLoS One. 2020 Feb 24;15(2):e0229332. doi: 10.1371/journal.pone.0229332 (PMC7039444; doi:10.1371/journal.pone.0229332)

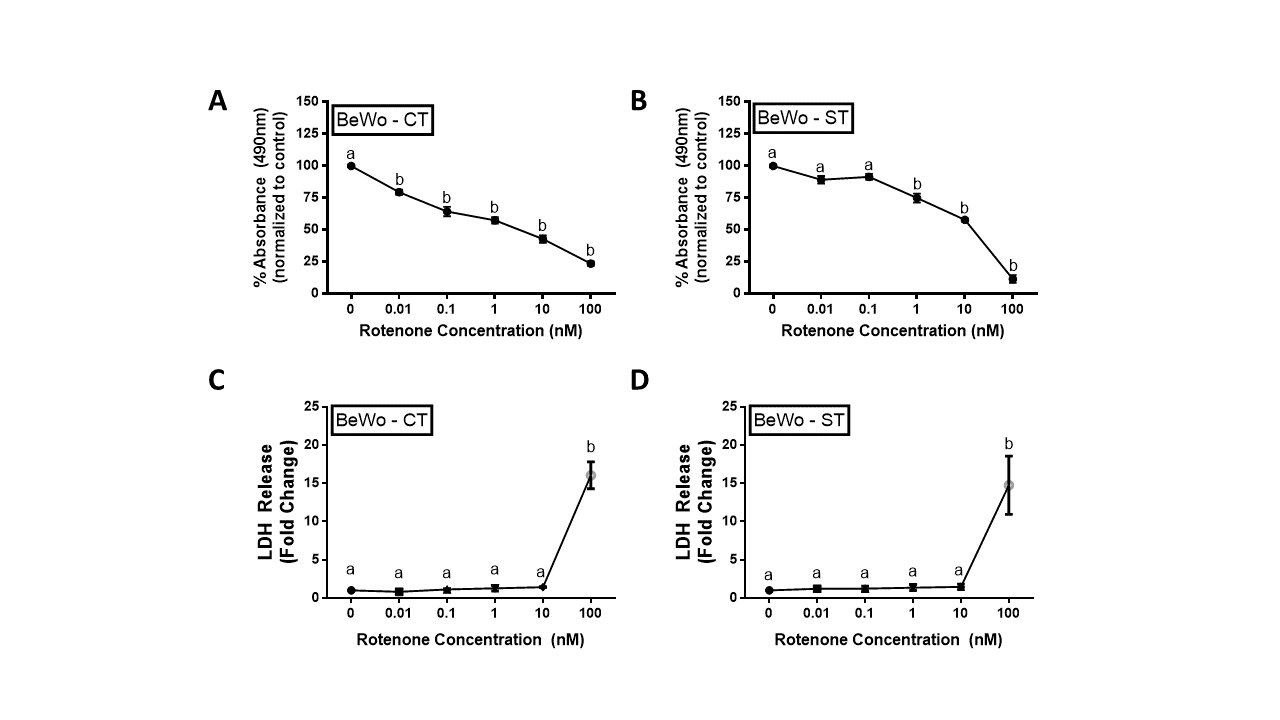

Supplement: S1 Fig — The cells were treated with rotenone as indicated. MTS data in panels A, B. LDH data in panels C, D. Each data point represents the mean ± S.E.M. of 3 replicates. Significant differences were determined by a one-way ANOVA followed by a Bonferroni post hoc test. Points with different letters differ significantly at P < 0.0001 (A-C) P < 0.001 (D) from control. Further experiments with rotenone were carried out by using the concentration that gave half of the maximum response, i.e. the point at which MTS values were reduced by 50%, thus successfully inhibiting RCC1 without causing overwhelming toxicity to the cells (C, D). (PNG) [file pone.0229332.s001.png]

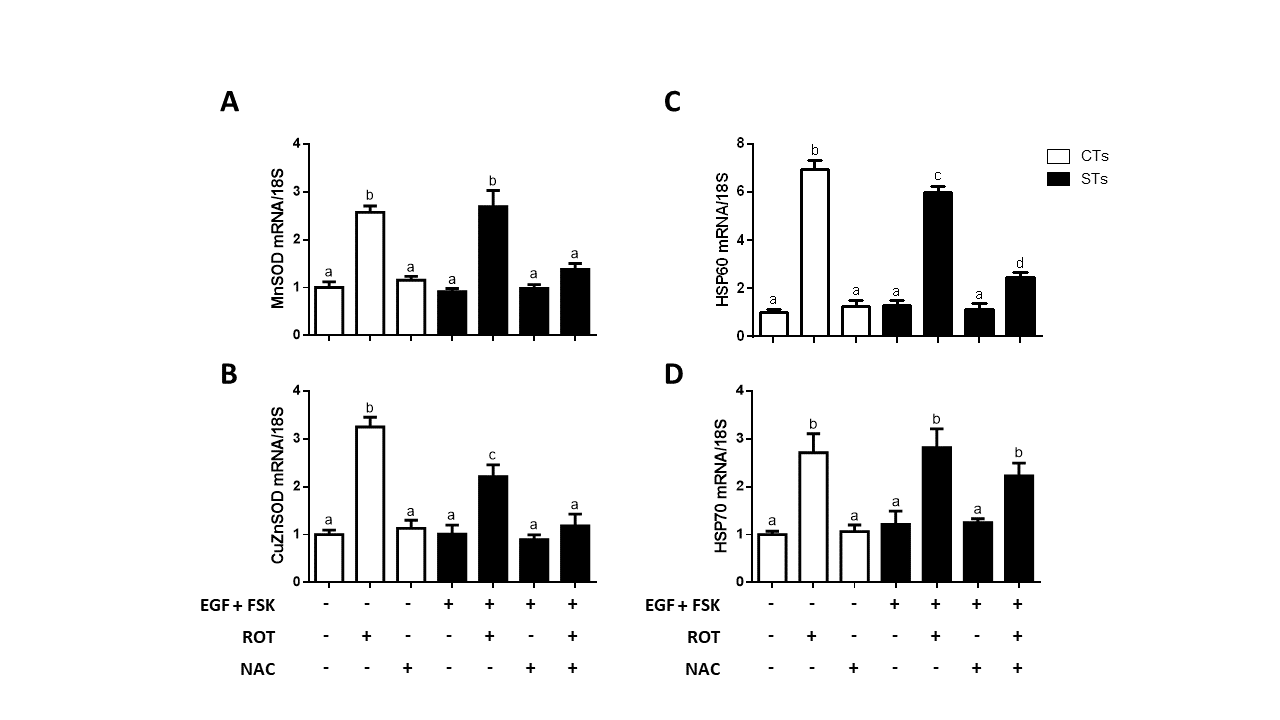

Supplement: S2 Fig — Total RNA was isolated from the cells and analyzed by RT-PCR (500 ng) with 18S used as the housekeeping gene. (A—D) Summary histograms of relative MnSOD (A), CuZnSOD (B), HSP60 (C), and HSP70 (D) mRNA expression in each treatment group normalized to 18S, then compared to the gene in the vehicle control group. Significant differences were determined by a one-way ANOVA, followed by a Bonferroni post hoc test. Data are presented as mean ± SEM, n = 3. Bars with different letters differ significantly at P < 0.0001 (A), P < 0.001 (B), P < 0.01 (C), P <0.05 (D). (PNG) [file pone.0229332.s002.png]
